# Unveiling the Expected: A Case Report of a Massive Congenital Lung Cyst in a 22-Year-Old Male

**DOI:** 10.7759/cureus.65183

**Published:** 2024-07-23

**Authors:** Nosagie Ohonba, Devon Thorpe, Yashini Gopal, Kiana Bhola, David Jordanovski

**Affiliations:** 1 Department of Internal Medicine, Overlook Medical Center, Summit, USA; 2 Department of Internal Medicine, Overlook Medical Center, New Jersey, USA

**Keywords:** cavitary lung lesions, cystic malformation, cpam, congenital cystic lung lesions, congenital lung malformations

## Abstract

Congenital pulmonary airway malformations (CPAMs) are predominantly identified prenatally or during infancy, with adult-onset cases being considered extremely rare. This case report describes a 22-year-old male who presented with hemoptysis and exertional dyspnea, leading to the diagnosis of CPAM. The patient had experienced small-volume hemoptysis for four years, which escalated to larger volumes and progressive dyspnea one week before hospital admission. A chest CT scan revealed a large 13.2 cm thin-walled cavitary lesion with an air-fluid level in the left lower lobe. The patient underwent left video-assisted thoracoscopic surgery (VATS) resection, which confirmed a CPAM originating from the left lower lobe. The postoperative recovery was uneventful, and the patient was symptom-free at follow-up. This case highlights the need to consider CPAM in the differential diagnosis of respiratory symptoms in young adults, even without congenital anomalies or predisposing factors. Early recognition and surgical intervention can lead to favorable outcomes.

## Introduction

Congenital pulmonary airway malformations (CPAMs) are typically identified prenatally or in infancy, with adult-onset cases being exceptionally rare [1]. CPAM results from the cessation of lung development during various stages of embryogenesis. Infants with this diagnosis can exhibit a wide range of severity, from being asymptomatic until later in life to experiencing respiratory distress during the neonatal period. Incidence data from large population registries suggests congenital lung cysts occur at rates ranging from 1 per 8,300 to 35,000 live births [1]. An estimated 80% of the lesions are recognized in the neonatal period; however, there are few case reports highlighting diagnosis in adulthood [2]. Adult patients may either be asymptomatic or exhibit symptoms such as recurrent infections, pneumothorax, shortness of breath, or hemoptysis [3]. We present the case of a 22-year-old male without prior significant medical history who presented with hemoptysis and exertional dyspnea, leading to the unexpected diagnosis of CPAM. The case highlights the unusual presentation of CPAM in adulthood and emphasizes the importance of considering this condition in the differential diagnosis of respiratory symptoms, even in individuals without a history of congenital anomalies.

## Case presentation

A 22-year-old male with no past medical history presented to the hospital with a two-day complaint of hemoptysis and exertional dyspnea. He had experienced episodic, small-volume hemoptysis over the previous four years, which was never investigated. However, within the week prior to presentation, he began having larger-volume hemoptysis and progressive dyspnea on exertion. He denied a recent fever, chest pain, respiratory infection, or coryzal symptoms. He had no history of serositis, pleuritis, synovitis, arthritis, or bleeding diathesis. His additional history was negative for recent medication use beyond naproxen, recent sick contacts, recent travel, or trauma. Family history was also negative for bleeding disorders or autoimmune diseases, and the patient did not use recreational drugs.

**Figure 1:**
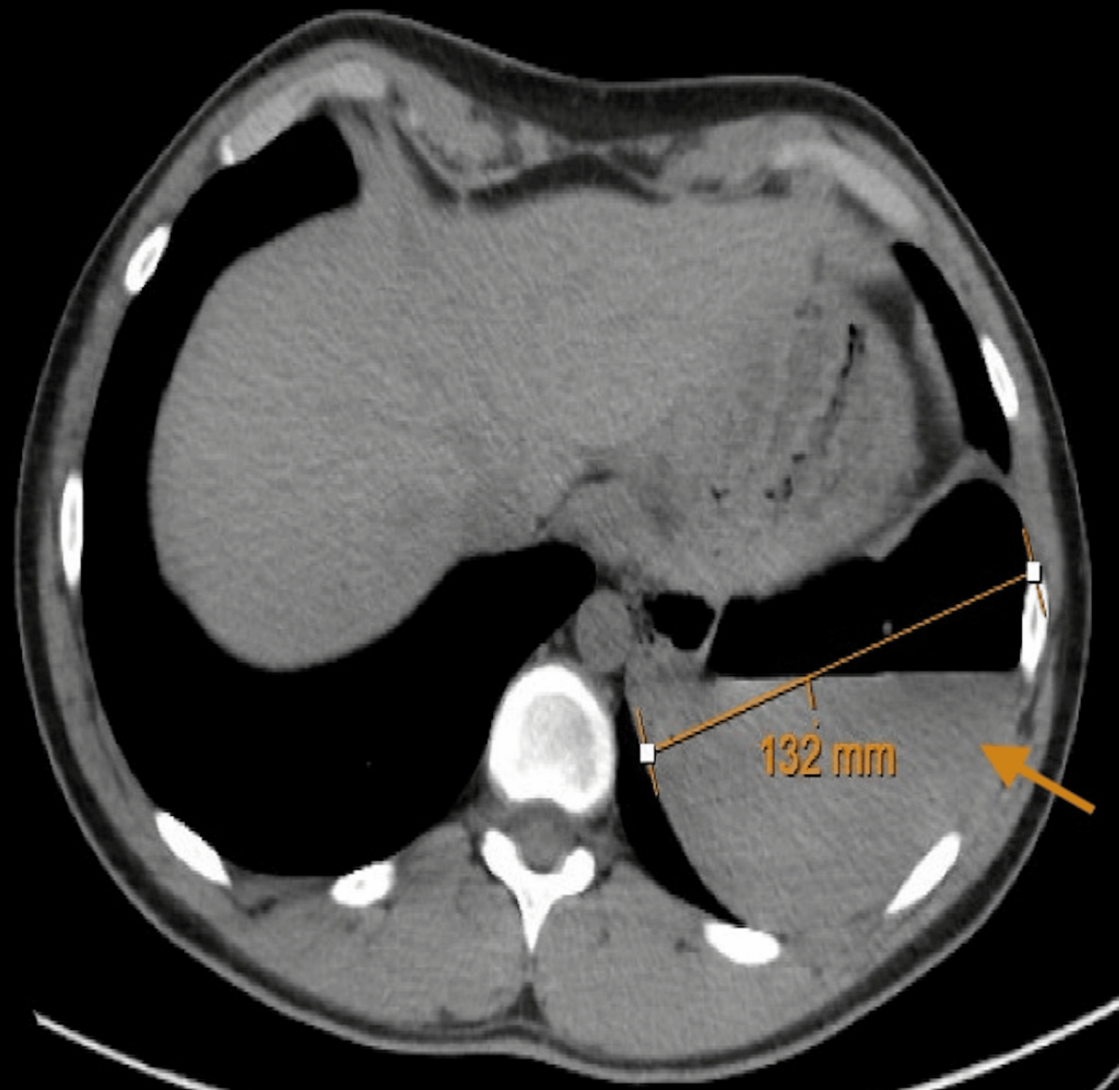
Axial CT scan of the chest showing a large 13.2 cm thin-walled cavitary lesion with an air-fluid level in the left lower lobe, as indicated by the arrow

**Figure 2:**
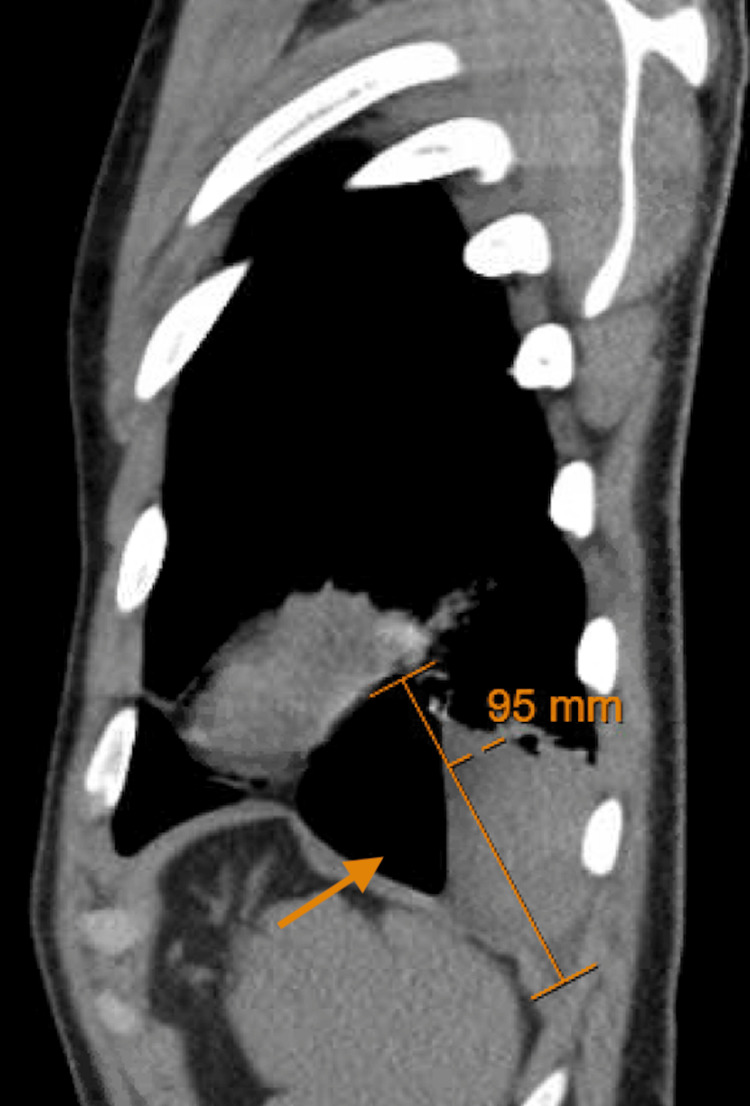
Sagittal CT scan of the chest showing a thin-walled cavitary lesion with an air-fluid level in the left lower lobe, as indicated by the arrow

Upon presentation to the hospital, his vitals remained stable, and he was oxygenating at 98% on room air without accessory muscle use or respiratory distress. His examination was unremarkable, and his chest was clear to auscultation. A chest CT scan with contrast revealed a large 13.2 cm thin-walled cavitary lesion with an associated air-fluid level, replacing most of the inferior segment of the left lower lobe, with atelectatic changes in the subjacent lung (Figure 1, Figure 2).

An extensive workup was performed, including tests for CRP, ESR, C3 complement, C4 complement, D-dimer, respiratory viral panel, hepatitis serologies, urine drug screen, *Cryptococcus *antigen, *Coccidioides *antibodies (IgG and IgM), and urine histoplasma antigen. All results were negative, except for mild anemia (Table 1).

**Table 1:** Laboratory values ESR, erythrocyte sedimentation rate

Test	Result	Reference value
Hemoglobin	12.6 g/dl	14.0-17.0 g/dl
WBC	7.27/nl	4.5-11.0/nl
Absolute neutrophil count	4.07/nl	2.00-6.60/nl
Absolute lymphocyte count	2.41/nl	0.70-3.70/nl
Eosinophil count	0.21/nl	0.00-0.20/nl
Platelets	260/nl	150-450/nl
CRP	3.7 mg/l	0.0-9.0 mg/l
ESR	<1 mm/hr	0-23 mm/hr
C3 complement	103.0 mg/dl	90-180 mg/dl
C4 complement	25 mg/dl	10-40 mg/dl
D-dimer	<0.30 ug/ml	0.00-0.49 ug/ml
HIV 1/2 antigen and antibodies	Undetectable	Undetectable
Procalcitonin	0.02 ng/ml	0.00-0.25 ng/ml
Cryptococcus antigen	Negative	Negative
Coccidioides antibodies (IgG and IgM)	Negative	Negative
Urine histoplasma antigen	Negative	Negative
Hepatitis B surface antigen	Negative	Negative
Hepatitis C viral antibodies	Negative	Negative
Respiratory viral panel	Negative	Negative

A diagnostic and therapeutic left video-assisted thoracoscopic surgery (VATS) resection was performed. Inspection confirmed that the cyst originated from the medial basilar aspect of the left lower lobe. The large hemorrhagic cyst was incised, and the blood was evacuated. The cyst was then decompressed, and a pathology specimen was obtained. An interval chest tube was placed and removed prior to discharge.

The patient fully recovered from the surgical procedure and remained symptom-free. The pathology confirmed the diagnosis of CPAM.

## Discussion

CPAM, previously known as congenital cystic adenomatoid malformation (CCAM), is a rare congenital anomaly of the lung characterized by cystic and adenomatous overgrowth of terminal bronchioles. While CPAM is typically diagnosed in utero or during infancy due to associated respiratory distress or infection, cases presenting in adulthood are exceedingly rare, as seen in this 22-year-old male without any significant past medical history [1].

CPAMs stem from aberrations in lung branching morphogenesis during fetal development [4]. These anomalies are believed to arise at various levels of the tracheobronchial tree and at different stages of lung maturation, potentially influenced by factors like in utero airway obstruction or atresia.

Incidence data from large population registries suggests congenital lung cysts occur at rates ranging from 1 per 8,300 to 35,000 live births [1]. Among CPAMs, large-cyst subtypes predominate, constituting approximately 70% of cases, or 2-8 per 100,000 live births [5]. Formerly termed CCAMs, CPAMs were classified into three major types based on cyst size and cellular characteristics, primarily bronchial, bronchiolar, or bronchiolar/alveolar duct cells [6-8].

The clinical presentation of CPAM is diverse. While many are identified through routine prenatal ultrasound, recent advancements have led to the detection of smaller lesions, often asymptomatic at birth. Roughly 25% of newborns with CPAM exhibit symptoms, a notable decrease compared to earlier reports [9]. Approximately three-quarters of infants with a prenatal CPAM diagnosis remain asymptomatic initially, with one-third diagnosed after the neonatal period.

In older children, CPAM may manifest as recurrent pneumonia, cough, dyspnea, cyanosis, or even spontaneous pneumothorax. Physical examination findings commonly include decreased breath sounds over the lesion, hyperresonance, and chest wall asymmetry [9]. In this case, the patient presented with a sudden onset of hemoptysis and exertional dyspnea, symptoms that are not specific to CPAM but rather suggestive of a variety of pulmonary conditions, including infections, neoplasms, or vascular abnormalities.

The definitive diagnosis of CPAM often requires histopathological examination of the resected specimen, as was performed in this case [10]. Surgical resection via VATS is the mainstay of treatment for symptomatic CPAM, aiming to alleviate symptoms, prevent complications such as infection or hemorrhage, and potentially reduce the risk of malignant transformation.

## Conclusions

While CPAM is typically diagnosed in infancy, it should be considered in the differential diagnosis of young adults presenting with respiratory symptoms and radiographic evidence of cystic lung lesions. Timely recognition and surgical intervention can lead to favorable outcomes, as demonstrated in this case. Our case highlights the rarity of CPAM presenting in adulthood, particularly with an unusual symptomatology of hemoptysis. Such late presentations underscore the importance of considering CPAM in the differential diagnosis of respiratory complaints, even in individuals without a history of congenital anomalies or predisposing factors.
